# In vivo efficacy of top five surveyed Ghanaian herbal anti-malarial products

**DOI:** 10.1186/s12936-017-1757-4

**Published:** 2017-03-04

**Authors:** Dennis Wilmot, Elvis Ofori Ameyaw, Daniel Amoako-Sakyi, Johnson Nyarko Boampong, Neils Ben Quashie

**Affiliations:** 1 Department of Biomedical and Forensic Sciences, University of Cape Coast, Cape Coast, Ghana; 2 Centre for Tropical Clinical Pharmacology and Therapeutic, School of Medicine and Dentistry, University of Ghana, Accra, Ghana; 3 Noguchi Memorial Institute for Medical Research, University of Ghana, Accra, Ghana

**Keywords:** Anti-malarial drug, *Plasmodium falciparum*, ICR mice, *Plasmodium berghei*, Chemosuppression, Herbal-preparations, In vivo

## Abstract

**Background:**

Anti-malarial herbal preparations (HPs) continue to enjoy high patronage in Ghana despite reports that the artemisinin-based combination therapy (ACT), the recommended first choice for treatment of uncomplicated malaria in the country, remains efficacious. A major issue with the use of these preparations is inadequate or unreliable data on their efficacy and quality. An assessment of the potency and quality of the most popular commercial anti-malarial HPs in Ghana was, therefore, carried out. The outcome of this investigation is herein discussed preceded by a short literature review of herbal medicines in Ghana.

**Methods:**

Using a questionnaire survey of 344 individuals in parts of Ghana, five of the most frequently used HPs were identified and selected for test of their efficacy and quality. The effect of the selected compounds on *Plasmodium berghei* in vivo was assessed using standard methods.

**Results:**

All five tested HPs (HP-A, HP-B, HP-C, HP-D and HP-E) showed chemo-suppressive activity against *P. berghei* in vivo. However the degree of parasites inhibition is significantly lower compared to the WHO-recommended artemether–lumefantrine combination (p < 0.05, 99.9% chemosuppression/activity, 28 days survival). Using the Solomon Saker’s Test, two of the preparations were found to contain chloroquine or compounds with chemical properties like that of chloroquine.

**Conclusion:**

Popular anti-malarial HPs used in southern Ghana were found to have chemo-suppressive properties. Intentional addition of chloroquine or SCs to these preparations in order to enhance their effectiveness has serious public health concerns as it may induce cross resistance to amodiaquine, one of the partner drugs in the recommended ACT for use in Ghana.

## Background

The global fight against malaria is multi-faceted, employing a number of approaches including vector control and effective case management. The use of the ACT as first-line treatment against uncomplicated malaria was recommended by the World Health Organization (WHO) in the mid-2000s and up to date, ACT has been used extensively in most disease endemic countries to manage the disease. In Ghana, artesunate–amodiaquine (AA) is the first choice, with artemether–lumefantrine (AL) and dihydroartemisinin-piperaquine (DHAP) as alternatives. The use of the ACT coupled with the implementation of other interventional measures has resulted in significant reduction in the incidence of malaria in Ghana lately. Despite this gain and reports that the ACT still remains efficacious in the country, commercial anti-malarial herbal preparations continue to enjoy high patronage in Ghana. This situation may be due to the fact that herbal medicine offers a cheaper alternative to allopathic medicines. Worldwide, it is estimated that over 1200 plant species are used for the treatment of malaria and fevers, and herbal preparations are potentially important sources of new anti-malarial treatments [1].

The practice of using herbal medicine to treat malaria in Ghana is indigenous and widespread. An ethno-botanical survey of plants used by indigenous households in the Dangbe West District of Ghana revealed that a total of about 30 different species of plants are used by the people to treat malaria [2]. This trend could be similar in the other 215 districts in the country. Indeed, Buabeng et al.[3] demonstrated that about 9% of Ghanaians depend on HPs for the treatment of malaria. Of particular mention is *Cryptolepis sanguinolenta,* also known as nibima, which has been demonstrated to be clinically efficacious against malaria [4]. Herbal tea formulations of nibima, trademarked as Phyto-Laria, have been shown to offer 93.5% cure rate in vivo with no signs of toxicity. Other herbal formulation reported to have significant antiplasmodial activity for malaria treatment in Ghana include *Tridax procumbens* [5], *Phyllanthus amarus* [5], *Theobroma cacao* [6], *Haematostaphis barteri* [7] and *Plumeria alba* [8].

Plants showing anti-malarial activity need to be examined further in order to identify and unearth the potential phytochemicals present that may be responsible for the activity. Pursuing this will make herbal medicine a route for anti-malarial drugs discovery. In spite of the health benefit of herbal products, these preparations could also be a potential death trap. For instance, if the claimed cure by the peddler is not true, the life of the consumer may be at risk as high number of parasites may build up and may results in severe disease condition. Another health risk is when the preparation contains a toxic component. Again, if the HPs have active ingredients whose chemical properties are similar to certain allopathic drugs, then there could be cross resistance to such drugs as a consequence of selection pressure. Adulteration of HPs with allopathic drugs in order to increase their potency has been reported [9]. Such practices could lead to parasites developing resistance to these allopathic drugs.

Whilst the Food and Drugs Authority (FDA) monitors the toxicity of the commercially available HPs, the efficacy of most of these preparations remains unverified. With a public health approach, this study, therefore, explored the potency of some selected HPs in Ghana.

## Methods

### Survey sites

**Fig. 1:**
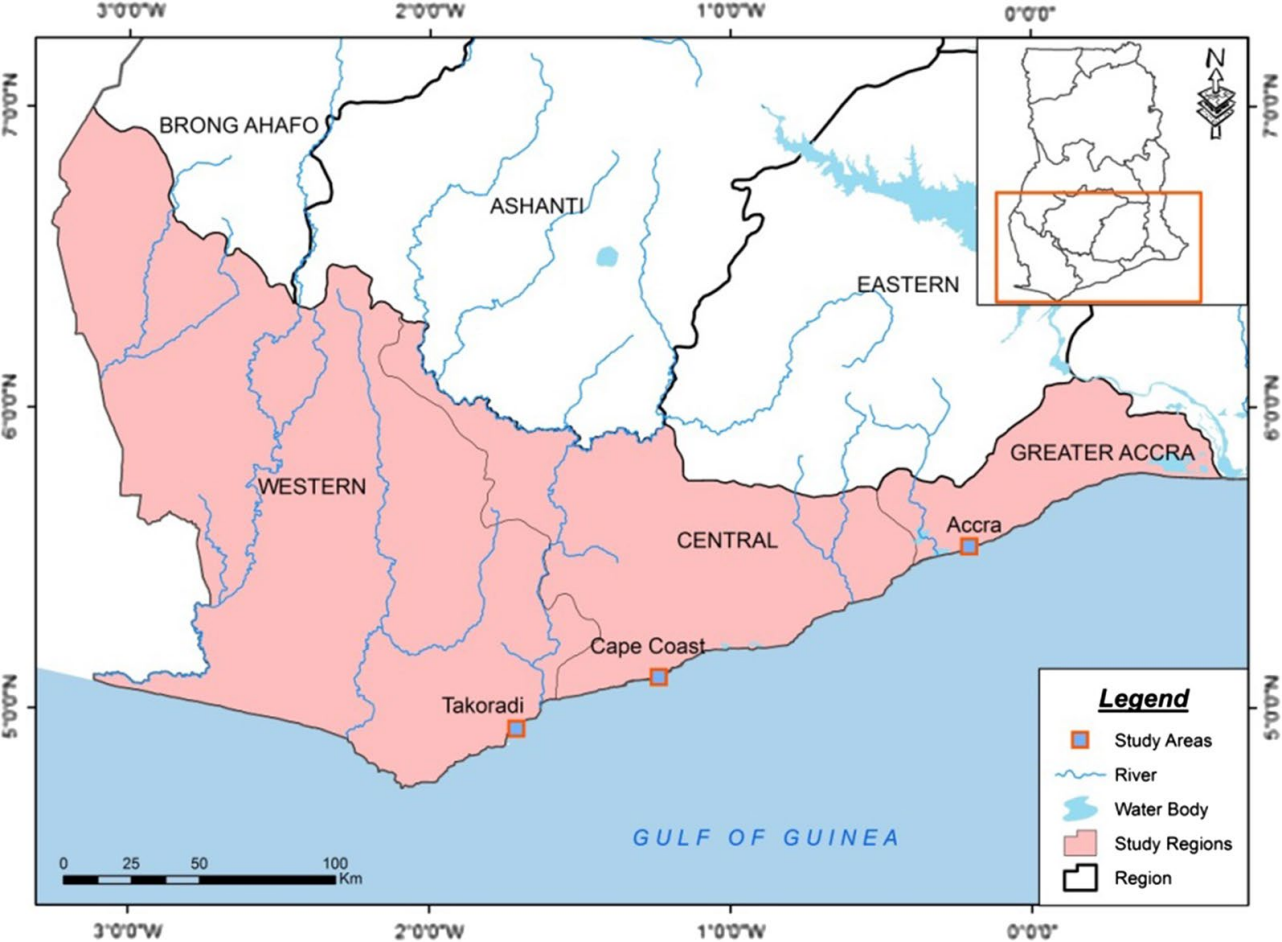
Map of Southern Ghana showing the sites for the survey. *Source* Remote Sensing and Cartographic Unit, University of Cape Coast, 2013

The survey was conducted in three Coastal regional capital cities shown in Fig. 1; Cape Coast (5° 6′ 0″ N/1° 15′ 0″ W) in the Central Region; Takoradi (4° 53′ 0″ N/1° 45′ 0″ W) in the Western Region; Accra (5° 33′ 0″ N/0° 13′ 0″ W) in the Greater Accra Region of Ghana. These sites were selected based on population density.

### Survey participants and sample size

This is an open gender-unbiased study. Individuals below the age of 18 years at the time of the questionnaire administration were excluded in the survey. Sampling at the street level was done randomly by interviewing every third individual or individuals from every second open shop. At the community level, every second house was chosen and the individuals in it were selected and interviewed. Based on the 2010 population census in Ghana, the sample size was estimated using the formula proposed by Cochran [10]. Three hundred and eighty-four (384) was the estimated sample size.

### Selection of herbal preparations and source of drugs

**Fig. 2:**
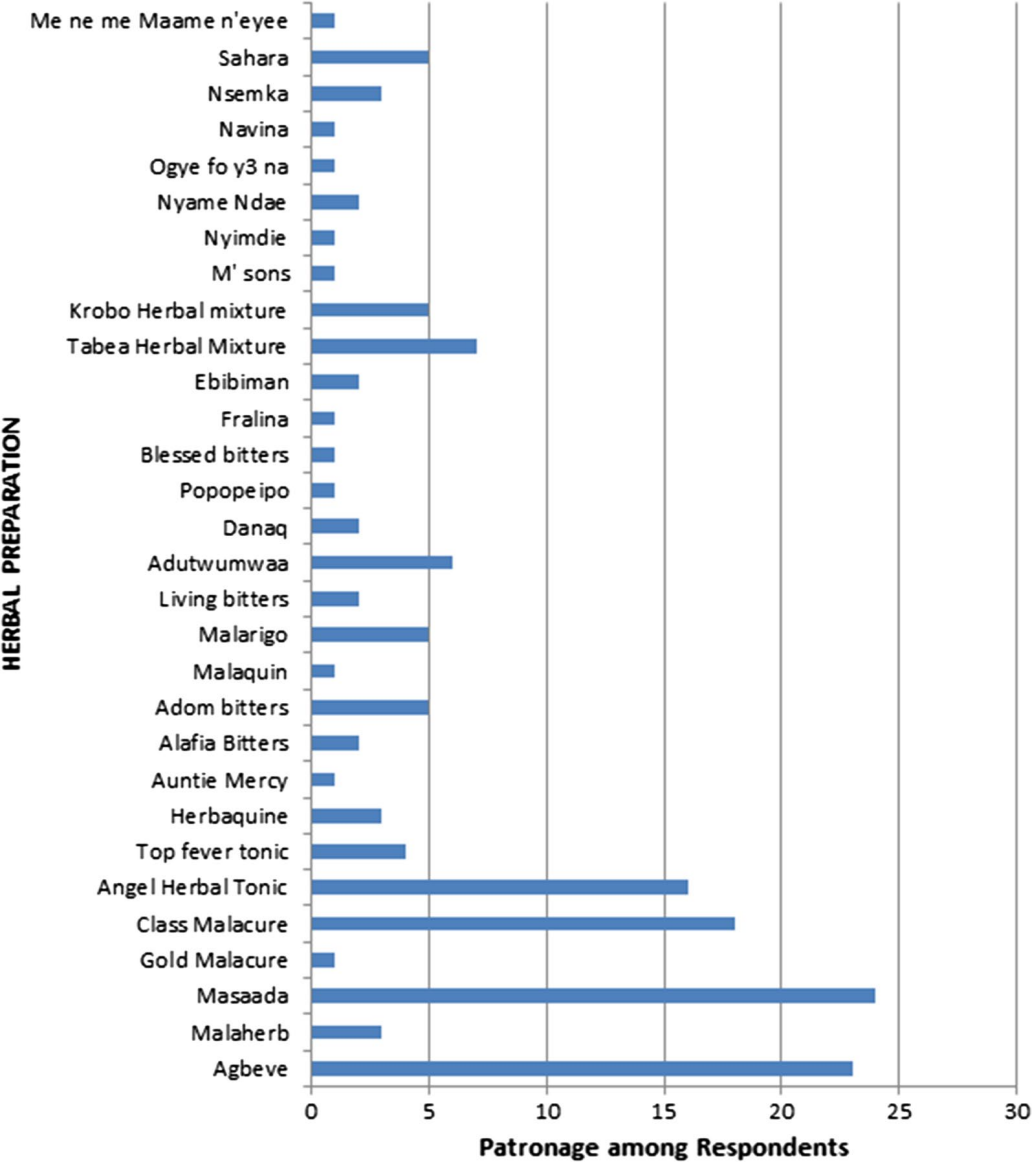
List of identified HPs being used to treat malaria in the surveyed areas

The herbal preparations mentioned by respondents in the questionnaire survey as their choice for treatment of malaria are shown in Fig. 2. The five most patronized HPs, as identified through the survey, were purchased from licensed pharmacy shops in and around the selected study sites. The five HPs were designated as: HP-A, HP-B, HB-C, HB-D and HB-E. Chloroquine-phosphate powder used as control in this study was a gift from Ernest Chemist. Artemether/lumefantrine (A–L), the designated standard allopathic anti-malarial drug was obtained from AJANTA Pharmaceutical Ltd (Mumbai, India).

### Rodent parasite used for in vivo tests

Male ICR mice (25–30 g) were housed in the animal facility of the Department of Biomedical and Forensic Sciences, University of Cape Coast (UCC). The animals were housed in groups of five in stainless steel cages (34 × 47 × 18 cm) with soft wood shavings as bedding. They were fed with normal commercial pellet diet (AGRICCARE, Kumasi). Water was given ad libitum, under laboratory conditions. The cages were kept in an environment of constant temperature (25–27 °C). All procedures and techniques used in these studies were in accordance with the National Institute of Health Guidelines for the Care and Use of Laboratory Animals [11]. All protocols used were approved by the UCC Ethical Committee.

The rodent parasite (*Plasmodium berghei* NK65) used in this study was provided by the Noguchi Memorial Institute for Medical Research, University of Ghana, Legon, Ghana. The parasites were initially stored at −70 °C in the laboratories of the Department of Biomedical and Forensic Sciences, University of Cape Coast until used.

### Evaluation of the anti-malarial activity of the HPs

The liquid HPs (1000 ml of each herbal product) were lyophilized with a freeze dryer. The anti-malarial effect of the selected HPs on *P. berghei* infection was evaluated using the method described by Al-Adhroey et al. [12]. Briefly, 85 male mice were each inoculated with 1 × 10^6^ *P. berghei* on the first day. Seventy-two hours after parasite inoculation, different groups of mice (five mice per group), were treated with one of three different doses, 10, 30 and 300 mg/kg p.o. of a chosen HP once per day. A group of five mice also received 4 mg/kg p.o. of artemether/lumefantrine (A–L). The negative control group, made up of also of five mice, was given 10 ml/kg p.o. normal saline. To determine the daily parasitaemia level, a drop of blood was collected from the tail of each mouse for preparation of thick and thin blood films on glass slide. The thin film was fixed with methanol and both films were stained with 10% Giemsa. The stained blood smears were then examined under the light microscope at 100× magnification. Parasitaemia was estimated as usual. The mean survival time of the mice in each treatment group was determined over a period of 28 days.

### Solomon Saker’s test for presence of chloroquine

The Solomon Saker’s test [13] was performed to ascertain adulteration of the HPs with chloroquine or compounds with chemical components similar to chloroquine. In brief, 1 ml nitrogen phosphate buffer and 0.2 ml tetrabromophenyl phtaline solution were dispensed into screw-cap conical centrifuge tubes. After addition of 2 ml HPs to the solution, the tubes were vigorously shaken for about 15 s and left to stand for about 15 min. The same was done for the positive control tubes which contain chloroquine at a concentrations of 2 μg/ml (low concentration control) and 4 µg/ml (high concentration control) respectively. A drug-free tube containing distilled water instead of drug was used as negative control. A yellowish-green colour of the organic layer indicates a negative test for CQ and metabolites; a red to purple colour of the organic layer indicates a positive result with the shade of colour determined by the concentration of CQ and metabolites present.

### Statistical analysis

GraphPad Prism for Windows version 4.03 (GraphPad Software, San Diego, CA, USA) was used for all statistical analyses. All data were expressed as mean ± SEM (duplicate measurement). The time-course curves were subjected to two-way (treatment × time) repeated measures analysis of variance (ANOVA) followed by Bonferroni’s post hoc test. *p* < 0.05 was considered statistically significant.

## Results

### Outcome of the questionnaire survey

From the questionnaire survey, a total of 31 different herbal medicines were mentioned to have been used. The names of the herbal preparations mentioned as used by the respondents to treat malaria are shown in Fig. 2. Five most patronized herbal preparations, as revealed by the survey, were investigated in this study. For the purposes of anonymity, the HPs were designated HP-A, HP-B, HP-C, HP-D, and HP-E.

### Effect of the herbal preparations on *P. berghei* infected mice

**Fig. 3:**
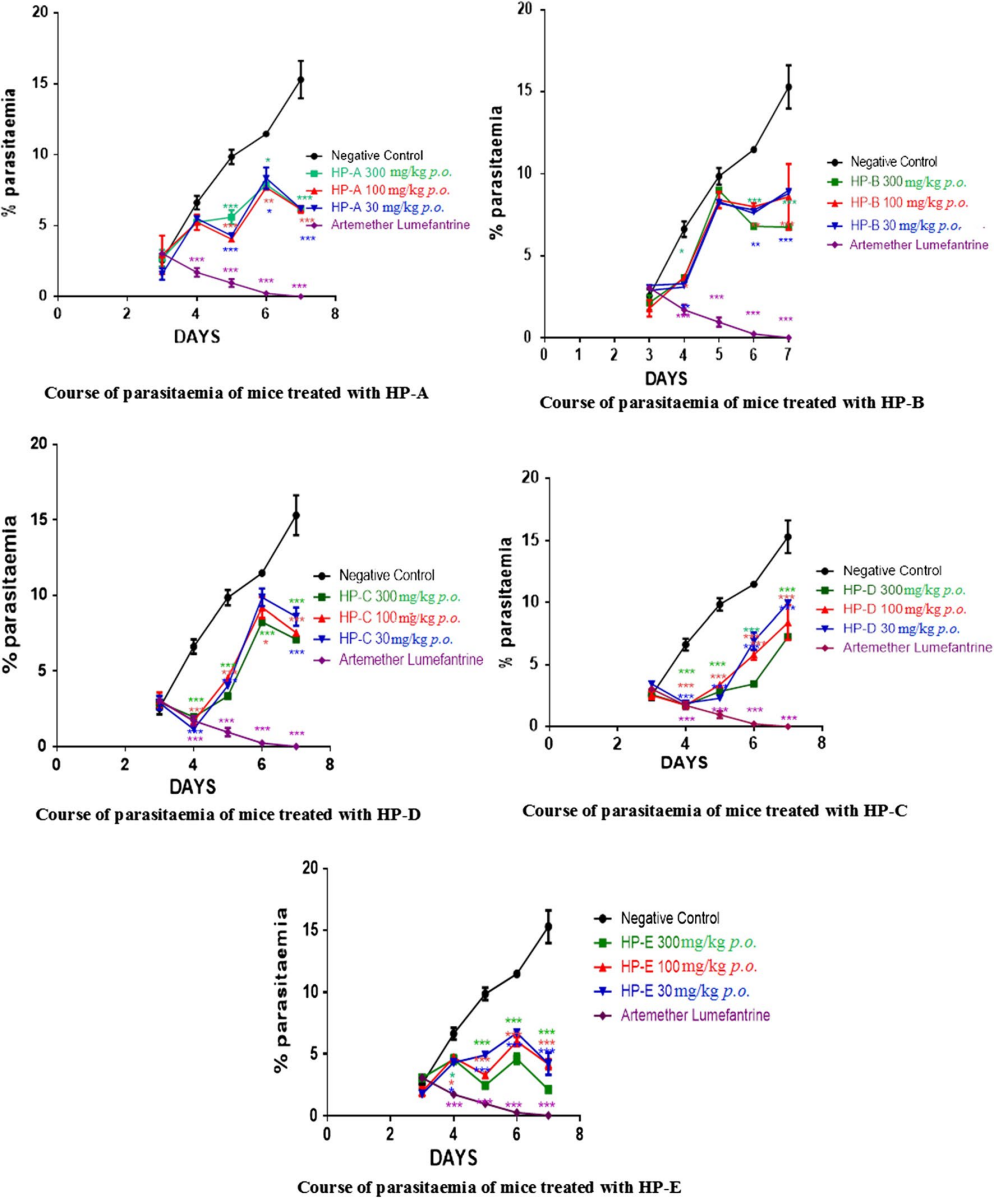
Time course of parasitaemia in mice treated with HPs. It shows the daily percentage parasitaemia of all three concentrations of HPs (300, 100 and 30 mg/kg body weight) compared to that of the negative control. Data is presented as mean ± SEM

Of the five selected herbal products investigated, only HP-A showed a dosage-independent chemo-suppression of *P. berghei*. However, almost all the test compounds were observed to be able to reduce parasitaemia significantly (p < 0.05) and consistently throughout the entire test period (Fig. 3). Two-way ANOVA testing followed by Bonferroni’s post hoc test revealed a significance difference between parasitaemia observed for the HPs and negative control. Chemo-suppression of the parasites by A–L was seen to be over 1.15 times greater than that of HP-E which was found to have the highest chemo-suppression activity.

### Chemo-suppression of parasitaemia and mice survival period

**Fig. 4:**
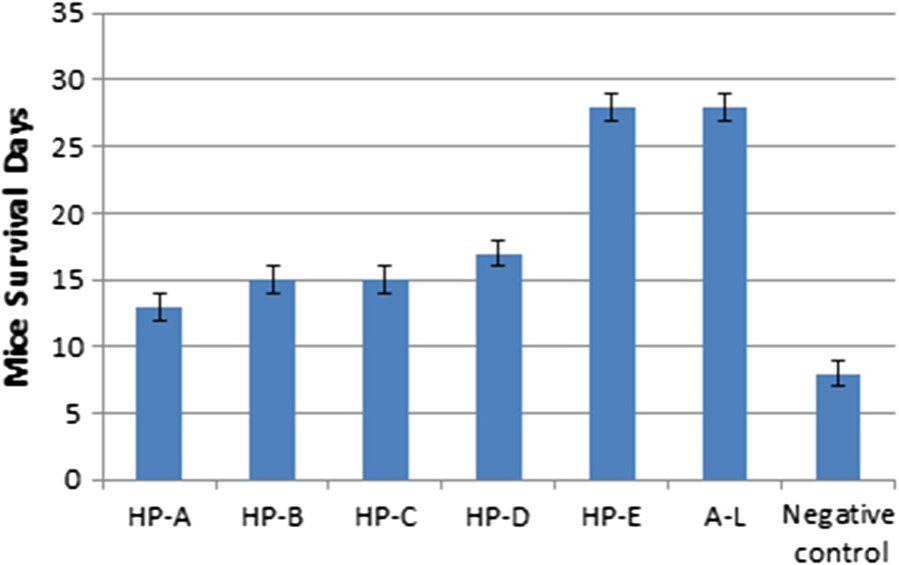
Mice survival period, shows the number of days survived by the mice after treatment with HPs or A–L compared with the negative control ±SEM

**Fig. 5:**
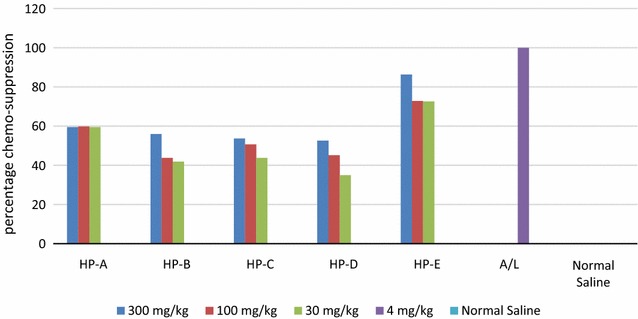
Chemo-suppression of parasitaemia, shows the percentage chemo-suppression of parasitaemia for each treatment

The mice survival period and percentage chemo-suppression of all the HPs as well as the control groups is shown in Figs. 4 and 5, respectively. Mice treated with HP-E survived up to day 28 of the test period. Mice given HP-A survived for the shortest number of days, 13 days, two less than those given Extracts B and C (15 days) and four less than those given HP-D (17 days).

### Solomon-Saker’s test for presence of chloroquine

In the Solomon Saker’s test, a light red colour was observed for the 2 μg/ml concentration of chloroquine and a red colour for the 4 μg/ml concentration of chloroquine. Two of the tested herbal preparations, HP-B and HP-C were found to exhibit colours similar to the positive control (high concentration, 4 μg/ml). This is indicative of the presence of high level of chloroquine or compounds with chemical properties similar to chloroquine in the two products.

## Discussion

For various reasons, a number of Ghanaians prefer herbal medicine over the ACT for the treatment of malaria, although available evidence indicates that the latter are still potent against *P*. *falciparum* infections in Ghana. Whether these herbal medicines are able to provide the claimed cure or protection against the disease remains largely unknown. This study therefore ascertained the efficacies of five of the most popular herbal preparations in Ghana and the implication of the findings on malaria control in Ghana is herein discussed.

Medicinal plants have been used in the treatment and prevention of malaria in various parts of the world and if found to have excellent anti-malarial properties, a herbal preparation stands a chance of becoming a candidate for further development on a similar scale as that of the quinine or artemisinin. The entire HPs investigated in this study exhibited chemo-suppression against *P. berghei* in vivo. Notably, HP-E was able to protect the mice against the parasites for up to 28 day post-infection making it worthy to receive attention for further future development. None of the test HPs could completely bring down parasitaemia in the first 2 days post-treatment. This could probably be due to low bioavailability of the HP in the mice or insufficient dosage among others.

Since the HPs are chemo-suppressive, their inability to completely clear parasites should be of concern to the malaria control program as it poses a potential danger to the health of the users due to anticipated persistency or delayed parasite clearance. Another danger is the likelihood of cross resistance if the HP shares similar chemical component with any of the recommended first line anti-malarial drugs, especially if the herbal medicine is not administered in the correct dosage. These observations made in this study are important as herbal medicine plays a significant role in the management of malaria in Ghana. Reports indicate that about 9% of the Ghanaian population use HPs for malaria treatment [3]. With the looming possibility of parasite resistance to the recommended ACT [14] coupled with difficulties in affordability and accessibility to these drugs in remote and poor areas in the country, anti-malarial herbal preparations would continue to be an important and sustainable source of malaria treatment in Ghana. It must however be emphasized that the use of these preparations must be closely monitored.

Solomon-Saker’s test indicated what could be the presence of chloroquine in the herbal preparation. This could be a case of intentional adulterations of the preparations in order to boost its efficacy. It is possible then, that chloroquine or compounds with chemical properties similar to chloroquine was the active ingredient responsible for the chemo-suppression exhibited by HP-B and HP-C. However the Solomon-Saker’s test has been shown to give false positive results for the anti-malarial drugs, quinine and proguanil [13]. It is, therefore, possible that quinine containing plants might have been used to prepare these two Solomon-Saker’s positive test compounds. Whatever the case may be the presence of chloroquine or compounds with chemical properties similar to chloroquine in these herbal preparations used by the Ghanaian public could foster the selection of chloroquine or quinine resistant clones in Ghana. For instance, Thompson et al. showed that *P. berghei* developed resistance to quinine administered in doses near the maximum amounts tolerated by mice [15]. This indicates the possibility of spontaneous or unintentional induction of parasite resistance to quinine or quinoline, by the use of an HP with similar chemical structure. The presence of these analogs in the HPs could probably explain the reasons for slow recovery of chloroquine sensitive clones in Ghana as reported by Afoakwah et al. [16]. Elsewhere, disuse of chloroquine resulted in the reversion of chloroquine sensitivity after a period [17].

Aside the possibility of adulterations with allopathic drugs or their analogs, the fact that these preparations were unable to completely clear parasites should be of public health concern as this could lead to the development of severe form of the disease. It is however assuring that the HPs showed some degree of anti-malarial activity. There is the need therefore to identify the active components in these HPs for further investigation and possible development to regulated drugs.

It must however be emphasized that since this study was performed using *P. berghei* in vivo, its comparison or extrapolation to field situation in Ghana, which is dominated by *P. falciparum* infections, is limited by differences in both the rodent host and the strain of malaria parasite. Nonetheless, results of this study should stimulate further investigations of Ghanaian HPs using other methods, such as the in vitro susceptibility test of *Plasmodium falciparum* to these preparations.

It is however interesting to note that in the course of preparation of this manuscript, Amoah et al. published the outcome of a similar study conducted in Ghana which may likely include some of the HPs assessed in this study [18]. Though the approach was similar, their work rather focused on assessment among others, of the gametocidal effects of herbal malaria products in the country. Whilst this work was performed in vivo, theirs was done in vitro. They demonstrated that some of the preparations indeed have gametocidal effect but showed little action against the asexual stages. This is interesting, since HPs with high gametocidal activity can help in the fight to reduce malaria transmission.

Put together, both studies have demonstrated that most of the HPs in Ghana possess a significant anti-malarial activity which justifies their use for the treatment of ‘fever’. This should open the way for a systematic interrogation of the chemical components of HPs present in Ghana in order to identify their active anti-malarial ingredients for isolation and further development.

## Conclusion

All five tested HPs showed various degree of chemo-suppressive activity against *P. berghei* in vivo. Two of the tested HPs have possibly been adulterated with chloroquine or compounds with chemical properties similar to chloroquine. Intentional adulteration of HPs with standard anti-malarial drugs should be of concern to stake holders in the health sector.

## Abbreviations

ACT: artemisinin combination therapy
A-L: artesunate+lumefantrine
CQ: chloroquine
HP: herbal preparation
HP-A: herbal preparation coded “A”
HP-B: herbal preparation coded “B”
HP-C: herbal preparation coded “C”
HP-D: herbal preparation coded “D”
HP-E: herbal preparation coded “E”
HPs: herbal preparations
ICR mice: imprinting control region mice
SEM: standard error of mean
SC: similar compound
WHO: World Health Organization

## References

[1] Bodeker G, Willcox M, Burford G, Willcox M, Bodeker G, Rasaoanaivo P (2004). An overview of ethnobotanical studies on plants used for the treatment of malaria. Traditional medicinal plants and malaria.

[2] Asasea A, Akwetey GA, Achel DG (2010). Ethnopharmacological use of herbal remedies for the treatment of malaria in the Dangme West District of Ghana. J Ethnopharmacol.

[3] Buabeng KO, Duwiejua M, Dodoo ANO, Matowe LK, Enlund H (2007). Self-reported use of anti-malarial drugs and health facility management of malaria in Ghana. Malar J..

[4] Bugyei K, Boye G, Addy M (2011). Clinical efficacy of a tea-bag formulation of *Cryptolepis sanguinolenta* root in the treatment of acute uncomplicated falciparum malaria. Ghana Med J..

[5] Appiah-Opong R (2011). Antiplasmodial Activity of Extracts of *Tridax Procumbens* and *Phyllanthus Amarus* in *in Vitro Plasmodium falciparum* Culture Systems. Ghana Med J..

[6] Komlaga G, Cojean S, Dickson RA, Beniddir MA, Suyyagh-Albouz S, Loiseau PM (2016). Antiplasmodial activity of selected medicinal plants used to treat malaria in Ghana. Parasitol Res.

[7] Boampong J, Karikari A, Ameyaw E (2015). *In vivo* antiplasmodial and in vitro antioxidant properties of stem bark extracts of *Haematostaphis barteri*. Asian Pac J Trop Biomed..

[8] Boampong JN, Ameyaw EO, Kyei S, Aboagye B, Asare K, Afoakwah R (2013). *In vivo* antimalarial activity of stem bark extracts of *Plumeria alba* against *Plasmodium berghei* in imprinting control region mice. J Parasitol Res..

[9] Debella A, Abebe D, Mudie K, Tadele A, Gebreegziabher A (2008). Qualitative laboratory analysis for the detection of conventional drugs in herbal preparations supplied by healers in major towns of Ethiopia. Ethiop J Health Dev..

[10] Cochran WG (1963). Sampling techniques.

[11] National Institute of Health (1996). Guidelines for the care and use of laboratory animals. Bethesda: Office of Science and Health Reports, Department of Health and Human Services; 1996.

[12] Al-Adhroey AH, Nor ZM, Al-Mekhlafi HM, Mahmud R (2010). Ethnobotanical study on some Malaysian anti-malarial plants: a community based survey. J Ethnopharmacol.

[13] Mount DL, Nahlen BL, Patchen LC, Churchill FC (1989). Adaptations of the Saker-Solomons test: simple, reliable colourimetric field assays for chloroquine and its metabolites in urine. Bull World Health Organ.

[14] Ajayi N, Ukwaja K (2013). Possible artemisinin-based combination therapy-resistant malaria in Nigeria: a report of three cases. Rev Soc Bras Med Trop.

[15] Thompson PE, Bayles A, Olszewski B, Waitz JA (1965). Quinine-resistant *Plasmodium berghei* in mice. Science.

[16] Afoakwah R, Boampong JN, Egyir-Yawson A, Nwaefuna EK, Verner ON, Asare KK (2014). High prevalence of PfCRT K76T mutation in *Plasmodium falciparum* isolates in Ghana. Acta Trop.

[17] Laufer MK, Takala-Harrison S, Dzinjalamala FK, Stine OC, Taylor TE, Plowe CV (2010). Return of chloroquine-susceptible falciparum malaria in Malawi was a reexpansion of diverse susceptible parasites. J Infect Dis.

[18] Amoah L, Kakaney C, Kwansa-Bentum B, Kusi K (2015). Activity of herbal medicines on *Plasmodium falciparum* gametocytes: implications for malaria transmission in Ghana. PLoS ONE.

